# Royal Dental Hospital of London

**Published:** 1902-03-22

**Authors:** 


### ROYAL DENTAL HOSPITAL OF LONDON.

### CHANGE OF SITE AND OF TITLE.

Though ladies were, in the advertisement convening it, specially invited to attend the annual general meeting of the Royal Dental Hospital of London, on Thursday last week, only two graced the lecture theatre with their presence. Mr. John Hampton Hale, who has succeeded Mr. Allen Stoneham as chairman of the managing committee, presided, the audience numbering some 14 governors.

He was sure they would all agree, the Chairman said, that the past year had been a satisfactory one, particularly to the members of the managing committee. The new hospital had been opened, and was in full work, and it had obtained a new name—the Royal Dental Hospital. It was quite certain that His Majesty would not have become their patron, and commanded the change of title, had he not been thoroughly satisfied as to the working of the charity. The hospital had now been rebuilt and fully equipped—not luxuriously, but everything that had been found necessary, both to the patients and to the staff, had been added to the appointments. As to the work, there had been 70,000 operations performed during the year, and a comparison of that figure with the 19,000 during 1874, when the hospital began operations, would show the progress made. The adverse aspect of affairs was that the expense of the building and equipment had amounted to something like £100,000; £18,000 had been realised for the old site, and they were still terribly in debt. Hearty thanks, however, were due to the Prudential Insurance Company, which had advanced them the money on mortgage in a liberal spirit, charging them only 3½ per cent., and allowing them to extend the repayment over a very long period—30 years. Very few people would have come forward in that way, because they did not like mortgages on places adapted only to one purpose, and it was clear that a hospital was useful for little else than the purpose for which it was built. They had £3,000 a year to provide for redemption and interest on the mortgage, in addition to £2,000 which they still owed to their builders, and another £3,000 for maintenance and expenses, making £6,000 a year in all. And as years went on the number of patients, and therefore their expenses, would surely increase. He trusted this money would be forthcoming, and particularly he desired to see a substantial increase in the number of annual subscribers. Legacies were a spasmodic and uncertain source of income, but if every governor would get at least one friend to become an annual subscriber the work of the committee would be far easier and its main difficulties overcome. Their income from rents was now about £1,000 a year, and this might possibly be increased in the current year to some extent. They had also another £500 from outside sources. Some of their friends wanted them to put in a lift, but it would cost £800 to do so and another 30s. a week to run it; and being in the nature of a luxury the committee felt they could not do it just yet at any rate. A special fund for the purpose had been suggested, but they felt that such a fund might deter those who contributed to it from giving to the general sustenance. He was sure that anyone who knew the working of the hospital knew the great benefit it was to the poor. No poor person who applied for its benefits was ever refused; they would like also to give them the gas free without orders from governors, but could not see their way to this at present.

Looking at their affairs broadly, they might be very proud at what had been achieved, but he would like to remind them that the whole of the credit of the work done should be attributed to Mr. Allen Stoneham, the late Chairman of the Committee of Management. Every member regretted his retirement, but they had elected him (Mr. Hale) to the post and he would do his very best to further the success of the institution. He moved the adoption of the report and accounts.

These were adopted, as were also resolutions re-electing the Committee of Management, with the addition of the names of Lord Llangattock, Mr. Arthur Hodgson, Mr. Nigel Smith, and Dr. F. P. Haviland; and the re-election of Mr. F. A. Bevan as treasurer.

